# Viral Cyclins Mediate Separate Phases of Infection by Integrating Functions of Distinct Mammalian Cyclins

**DOI:** 10.1371/journal.ppat.1002496

**Published:** 2012-02-02

**Authors:** Katherine S. Lee, Andrea L. Suarez, David J. Claypool, Taylor K. Armstrong, Erin M. Buckingham, Linda F. van Dyk

**Affiliations:** 1 Department of Microbiology, University of Colorado Denver, Anschutz Medical Campus, Aurora, Colorado, United States of America; 2 Department of Immunology, University of Colorado Denver, Anschutz Medical Campus, Aurora, Colorado, United States of America; Emory University, United States of America

## Abstract

Gammaherpesvirus cyclins have expanded biochemical features relative to mammalian cyclins, and promote infection and pathogenesis including acute lung infection, viral persistence, and reactivation from latency. To define the essential features of the viral cyclin, we generated a panel of knock-in viruses expressing various viral or mammalian cyclins from the murine gammaherpesvirus 68 cyclin locus. Viral cyclins of both gammaherpesvirus 68 and Kaposi's sarcoma-associated herpesvirus supported all cyclin-dependent stages of infection, indicating functional conservation. Although mammalian cyclins could not restore lung replication, they did promote viral persistence and reactivation. Strikingly, distinct and non-overlapping mammalian cyclins complemented persistence (cyclin A, E) or reactivation from latency (cyclin D3). Based on these data, unique biochemical features of viral cyclins (e.g. enhanced kinase activation) are not essential to mediate specific processes during infection. What is essential for, and unique to, the viral cyclins is the integration of the activities of several different mammalian cyclins, which allows viral cyclins to mediate multiple, discrete stages of infection. These studies also demonstrated that closely related stages of infection, that are cyclin-dependent, are in fact genetically distinct, and thus predict that cyclin requirements may be used to tailor potential therapies for virus-associated diseases.

## Author Summary

Many viruses encode homologs of human oncogenes, including the gammaherpesvirus viral cyclin genes. These viruses cause lifelong infection associated with chronic diseases, including malignancies, which are exacerbated in immune deficiency. The conserved viral cyclins were first recognized nearly two decades ago, and despite extensive interest and study, their essential features for virus infection and disease have been elusive. We used a mouse model of these viruses to make recombinant viruses with viral or human cyclins knocked into the endogenous locus. We then determined the requirements for cyclins by genetic complementation in three distinct viral cyclin dependent aspects of infection. We report that the viral cyclins of different gammaherpesviruses are able to support all three stages of infection. However, none of the human cyclins can, and instead comprise distinct complementation groups that are functional in non-overlapping aspects of infection. We showed that gammaherpesvirus encoded cyclins are functionally conserved, and that their essential unique property is the assimilation of the functions of distinct mammalian cyclins within a single multifunctional gene. Finally, in dissecting the requirements for viral cyclins during gammaherpesvirus infection, we demonstrated that related stages of infection are genetically separable and therefore may be susceptible to specific therapeutic manipulation.

## Introduction

Gammaherpesviruses are oncogenic viruses that establish lifelong infection of the host. Primary gammaherpesvirus infection of healthy adult hosts results in an acute stage of lytic virus replication which is then cleared, with lifelong latent infection established primarily in B lymphocytes. A transient mononucleosis-like stage is associated with establishment of latent infection with Epstein Barr virus (EBV) and the murine gammaherpesvirus 68 (gHV68). The latent stage of infection is controlled by an active immune response, and immune deficient hosts suffer increased virus reactivation from latency and persistent infection (evidenced by ongoing production of infectious virus), both of which are associated with disease. Viral cyclin genes are conserved among gamma-2-herpesviruses, including the human Kaposi's sarcoma-associated herpesvirus (KSHV), and Epstein Barr virus (EBV), a closely related human gammaherpesvirus, uses positional homologs to up regulate expression of host D-type cyclins. Cyclins are the regulatory partners of the catalytic cyclin dependent kinases (cdks), which together regulate cellular DNA replication and cell division. Viral cyclins share the greatest sequence similarity to one another and to mammalian D-type cyclins, yet are functionally most similar to mammalian cyclins A and E [1]–[4]. Relative to mammalian cyclins, the viral cyclins confer increased kinase activity and demonstrate broader cdk binding and substrate specificity, as well as increased resistance to cyclin-dependent kinase inhibitors [5]–[10]. The viral cyclin (v-cyclin) protein of the mouse model gHV68 is abundantly expressed in lytic virus replication and in reactivation from latency [11], and v-cyclin transcript is also detected in latently infected B cells [12]. The first gammaherpesvirus viral cyclin gene was described in 1992 [13], since which time numerous activities of the viral cyclins have been discovered and proposed as important in gammaherpesvirus pathogenesis. However, to date, no study has addressed whether the unique biochemical features of the v-cyclin are essential to promote infection or if mammalian cyclins, with more restricted activities, are capable of promoting infection. This issue is particularly important given the increasing evidence that mammalian cyclins have an unexpected degree of plasticity and redundancy in promoting cell cycle progression [14] [15], yet specific cyclins are required for cell- or tissue-specific functions [16], [17]. The emerging picture of the mammalian cyclins in cell cycle, development and cancer present a compelling case for understanding the specific activities of the unique viral cyclins.

While extensive biochemical characterization of viral cyclins revealed multiple unique characteristics of viral cyclins relative to mammalian cyclins when expressed in isolation, the precise function of the viral cyclins in the context of virus infection has only more recently been elucidated. In transgenic studies in which the viral cyclins are constitutively expressed in mice, both the gHV68 and KSHV viral cyclins are tumorigenic [11], [18]. This observation, coupled with the known cell cycle promoting effects of the viral cyclins and viral cyclin expression in some gammaherpesvirus associated tumors, initially lead to a focus on the oncogenic effects of the viral cyclin during infection. However, given that gammaherpesvirus infection in healthy individuals rarely induces malignancy, the viral cyclin is very likely to have roles in promoting viral infection and pathogenesis. To rigorously assess the genetic contribution of the viral cyclin in the context of virus infection and gammaherpesvirus pathogenesis, we have made extensive use of the gHV68 mouse model and have now shown that the v-cyclin of gHV68 plays a critical role in several distinct aspects of virus infection. We demonstrated that virus production in acute pulmonary infection is dependent on the v-cyclin [19]. Additionally, we noted a dramatic decrease in the survival of persistently infected endothelial cells upon infection with v-cyclin-deficient virus [20]. Finally, we and others observed a profound defect in viral reactivation from latency in the absence of the v-cyclin [21], [22]. The requirement for the v-cyclin is manifested in many disease states, that is, the v-cyclin-deficient virus is attenuated in lethal pneumonia [19], arteritis [23] and chronic pulmonary disease [24], chronic mortality in immune deficient mice [25], and in atypical lymphoid hyperplasia [26] found in immune deficient mice and pathologically similar to EBV-induced post-transplant lymphoproliferative disease. In contrast, the v-cyclin is dispensable for viral replication, the establishment of latency [22] and the development of pulmonary lymphoma in immunodeficient mice [27].

To rigorously dissect the essential cyclin feature(s) required for the v-cyclin during virus infection, we generated a panel of recombinant viruses in which the v-cyclin of gHV68 was precisely replaced with the viral cyclin of KSHV (k-cyclin) or with multiple different mammalian cyclins. By testing the capacity of different viral and mammalian cyclins to substitute for the function(s) of the endogenous v-cyclin of gHV68 in known v-cyclin dependent parameters, we determined that the viral cyclins of gHV68 and KSHV were able to interchangeably fulfill all v-cyclin dependent parameters of infection. On the other hand, analysis of viral recombinants expressing mammalian cyclins revealed varying capacity to support v-cyclin dependent stages of infection. Unexpectedly, distinct and non-overlapping cyclins were capable of functioning in different stages of infection, an observation which allowed us to genetically separate reactivation from latency and viral persistence. In total, these studies demonstrate that the viral cyclins are uniquely multifunctional and mediate their complete function by possessing properties of multiple mammalian cyclins.

## Results

### Generation and characterization of recombinant cyclin viruses

**Figure 1:**
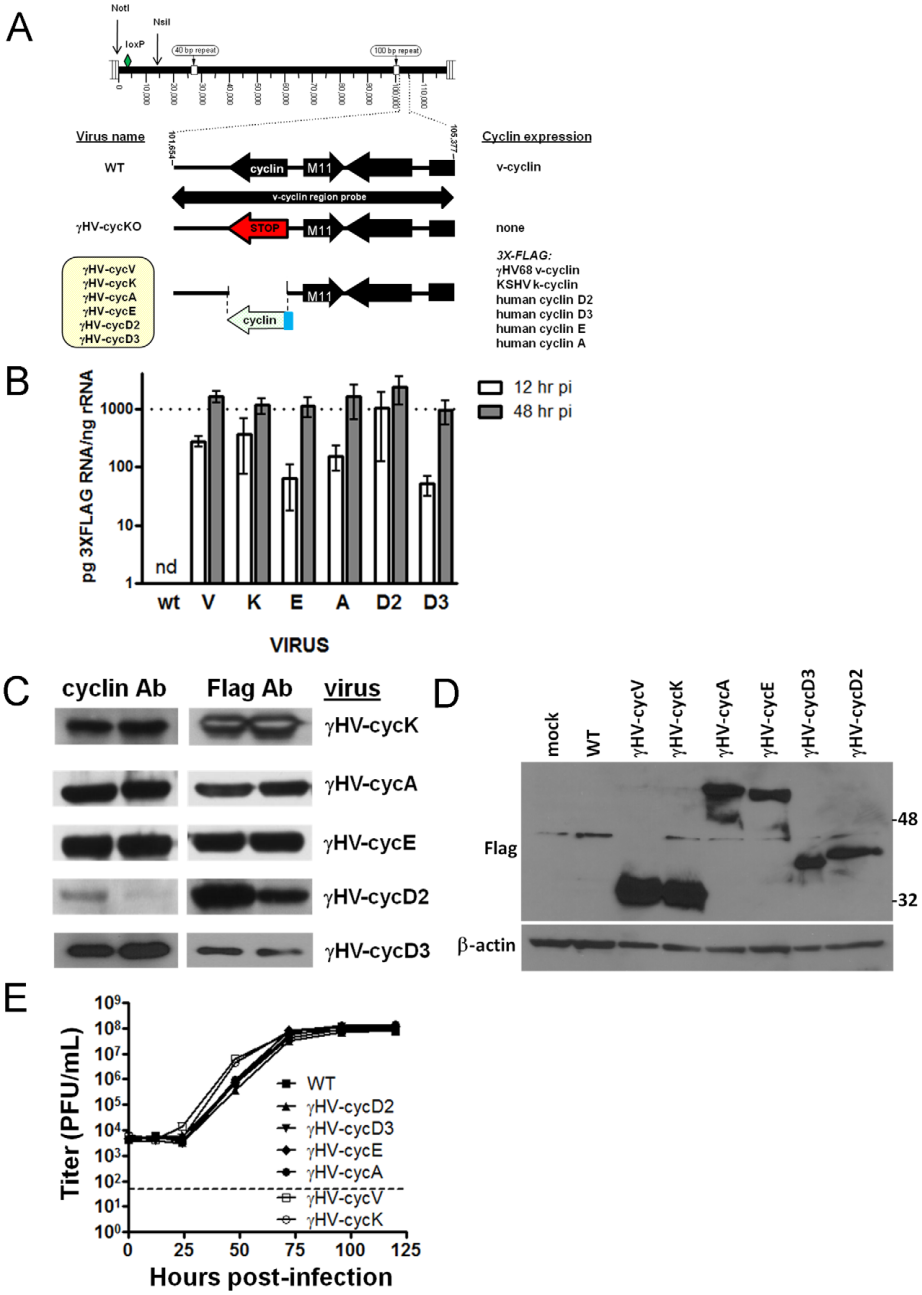
A panel of recombinant viruses express various cyclins under control of a uniform expression cassette. (A) Schematic representation of the recombinant viruses generated using BAC recombination. Genome coordinates of the v-cyclin ORF (bp 103181 to 102426) are based on the gHV68 WUMS sequence. The diamond denotes the location of the single 34 bp loxP site. The N-terminal 3x-FLAG epitope tag is shown as a blue rectangle. The cyclin region probe spans bp 101654 to 105377, and the left end probe spans bp 11100 to 14026. At the left end of the viral genome are the locations of the *Not*I and *Nsi*I sites used to diagnose possible deletions in this region of the genome. (B) Quantitative RT-PCR of RNA isolated from 3T12s infected for 12 (n = 2) and 48 (n = 3) hours with WT and recombinant cyclin viruses. Values are mean ± SD. One way ANOVA Bonferroni's Multiple Comparison Test determined no statistical differences between the samples. (C) Recombinant cyclin viruses express specific cyclin proteins. Lysates from 3T12s infected with two independent clones of recombinant cyclin viruses (designated at the right) for 24 hours were transferred and probed with the antibodies indicated above blots. The same number of cell equivalents was used and the data is representative of multiple experiments. (D) Recombinant cyclin viruses express each cyclin protein abundantly during lytic infection of 3T12 fibroblasts. Lysates from 3T12s infected for 24 hours were transferred and probed with antibodies to beta-actin, followed by the Flag epitope. 10 µg of lysate was loaded per lane and data is representative of multiple experiments. (E) Multistep replication of recombinant cyclin viruses. 3T12 cells were infected with recombinant cyclin viruses expressing viral or mammalian cyclins at a MOI of 0.05 PFU/cell and harvested at 0, 12, 24, 48, 72, 96 and 120 hours post-infection. Both cells and supernatants were analyzed by plaque assay. Data shown are from two independent infections, from which at least three plaque assays were performed per virus, with each sample being measured in triplicate. Mean ± SEM are shown. Dotted line indicates the limit of detection of the assay (50 PFU).

We generated a complete panel of recombinant viruses to genetically test cyclin requirements in promoting gammaherpesvirus infection (Figure 1A and S1, Table S1). Using bacterial artificial chromosome mediate mutagenesis, we generated six viral recombinants in which different viral or mammalian cyclins precisely replaced the endogenous cyclin gene. This method placed different cyclins under control of the endogenous v-cyclin promoter and viral polyA signal to faithfully recapitulate the transcriptional regulation of this gene. To facilitate uniform and sensitive detection of cyclin expression among these recombinant viruses, a 3x-FLAG epitope tag was fused to the amino terminus of each cyclin [28]–[30]. The cyclins included in this recombinant panel were based on similarity in either sequence or function to the v-cyclin: 1) the gHV68 v-cyclin, 2) the viral cyclin of KSHV (k-cyclin), 3) the mammalian cyclins D2 and D3, based on sequence similarity [6], [10], [31] and their predominant expression in lymphocytes [32], the major reservoir for gHV68 latency, and 4) the mammalian E and A cyclins based on structural and functional similarity [33]. Quantitative analysis of virally expressed cyclin mRNAs, via the shared 3x-FLAG sequence, demonstrated similar RNA expression of all 3x-FLAG tagged cyclins during virus infection at 12 and 48 hours post-infection. Further, all 3x-FLAG-cyclin RNAs were expressed at low levels 12 hours post-infection, and were abundant by 48 hours post-infection (Figure 1B), consistent with the early-late gene kinetics previously established for the gHV68 v-cyclin [11]. These data demonstrated that the FLAG-tagged cyclins are equivalently transcribed during virus infection. Viral cyclin protein expression during infection was detectable by immunoflourescence at 12 and 24 hours post-infection (Figure S2C), and demonstrated a similar and primarily nuclear/perinuclear pattern. Specificity of cyclin protein expression from each recombinant virus was verified by western analysis of independent duplicate infections using both FLAG- and cyclin-specific antibodies (Figure 1C), and abundant expression of each cyclin protein was demonstrated during infection of fibroblasts (Figure 1D) and endothelial cells (Figure S2A). Because viral and mammalian cyclins are presumed to function via binding of cellular cdks, we performed kinase interaction analyses for each of these viruses at 24 hours post-infection of two different cell types. Infection of both fibroblasts and endothelial cells demonstrated the expected interaction partners, with the viral cyclins binding cdks more efficiently than their cellular counterparts (Figure S2B). Notably, the cdks associated with the viral cyclins were distinct from each other, and neither of the viral cyclins shared a common interaction profile with any of the mammalian cyclins during virus infection. Variation in relative protein abundance and in kinase binding of the 3x-FLAG cyclins is consistent with known differences in protein stability and partner preferences, and notably did not correspond to function during virus infection in subsequent studies. Finally, we previously showed that the v-cyclin is not required for virus replication in vitro [22]. To ensure that insertion of other cyclin genes did not alter viral replication, we compared virus replication of WT virus with the panel of cyclin recombinants in multiple cycles of replication and found indistinguishable replication among these viruses (Figure 1E). Thus, replacement of the v-cyclin of γHV68 with other cyclin genes does not alter virus replication in vitro.

### Viral cyclins are uniquely competent in virus production and pathogenesis in acute pulmonary infection

**Figure 2:**
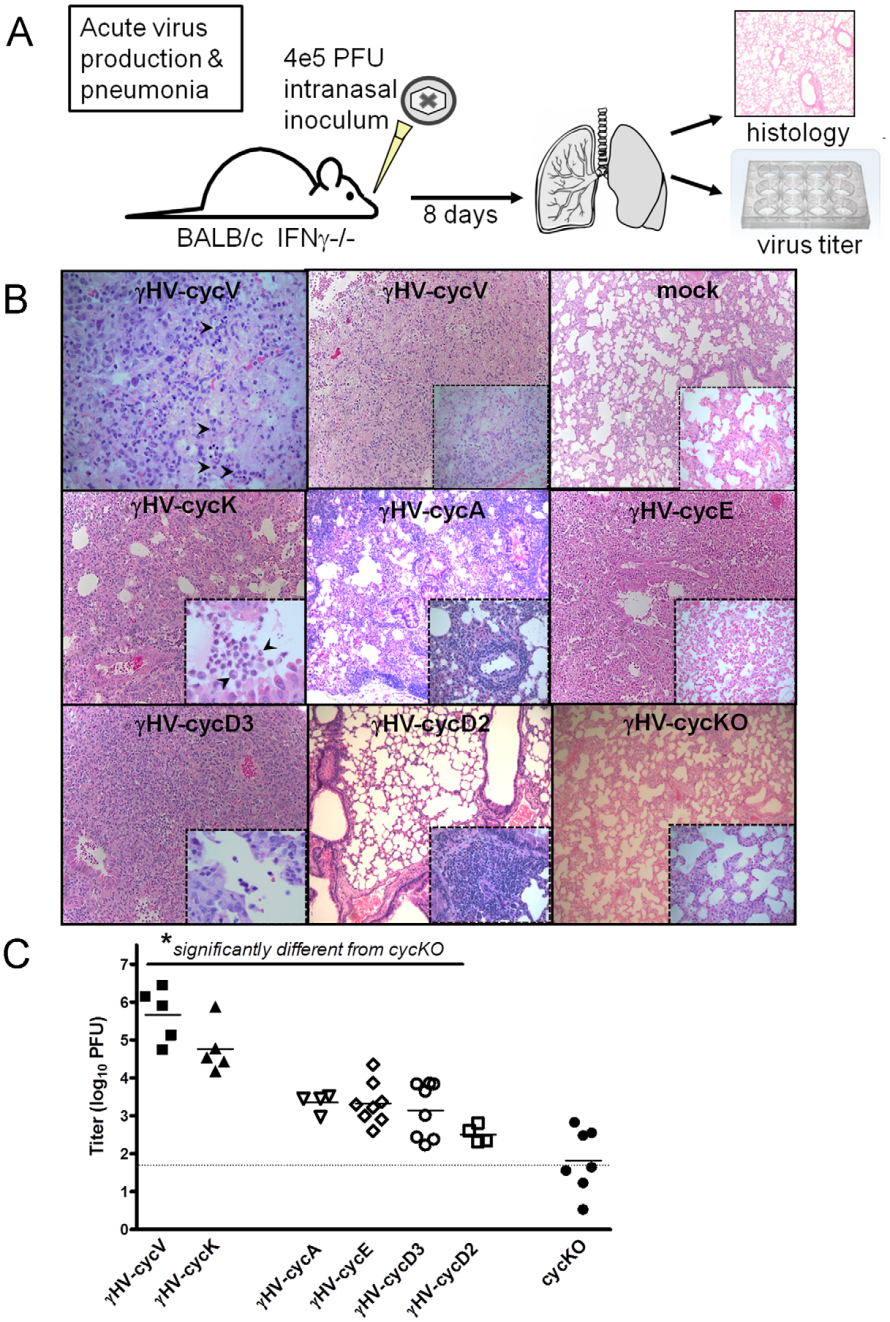
Acute defect in virus production is restored by viral cyclins. (A) Schematic representation of infections for lung titers and pathology in IFN-g-/-mice. Five mice were included in each group. (B) Histological analyses of lung tissues from mock, gHV-cycKO, gHV-cycV, gHV-cycK, gHV-cycA, gHV-cycE, gHV-cycD3 and gHV-cycD2-infected mice. Representative H&E images show clear and healthy airspaces in mock infected image and examples of neutrophil infiltrates indicated by arrowheads. Insets and upper left panel magnification ×40, all other panels magnification ×10. (C) Viral titers in the lungs were determined by plaque assay. Limit of detection is indicated by dotted line. Titers were statistically significant (*p<0.05) when compared to gHV-cycKO, as determined by a one-way ANOVA test.

As we recently reported, infection of immunodeficient mice with gHV-cycKO virus resulted in a significant defect in acute virus production in the lung and failed to cause the lethal pneumonia that results from WT infection and WT levels of acute virus production [19]. Therefore, to investigate the cyclin requirements for acute virus production and lung pathology, we infected IFN-g-/- mice with the panel of recombinant cyclin viruses. We tested gHV-cycK, the cyclin with the greatest overall similarity to the v-cyclin, gHV-cycD3 and D2 for sequence similarity and cell type relevance, and gHV-cycA and E for functional similarity. We infected IFN-g-/- mice with the recombinant cyclin viruses for 8 days, previously identified as the time at which virus titer and lung pathology differed most between WT and gHV-cycKO infection (Figure 2A) [19]. The severity of pathology, or acute pneumonia, was most profound following infection with the gHV-cycV, with similar morphology in the gHV-cycK-infected lungs (interstitial and airway edema, hypercellularity, tissue condensation and severe inflammatory infiltrates marked by neutrophils; Figure 2B), and mice in these groups demonstrated hunched posture and ruffled fur at time of sacrifice. Less severe pathology was observed in lungs infected with viruses expressing mammalian cyclins (inflammatory cell infiltrates and edema primarily surrounding vessels; Figure 2B) and these mice did not show physical symptoms. Similarly, virus production in acute lung infection was fully restored by the viral cyclins, whereas all viruses expressing mammalian cyclins were impaired relative to those expressing the viral cyclins (all statistically significant relative to gHV-cycV, p≤0.05), but did partially restore acute lung titers relative to gHV-cycKO (Figure 2C). These data suggest that mammalian cyclins have only a modest ability to function in viral infection and that the viral cyclins are unique in their ability to facilitate viral pathogenesis in lungs at early times post-infection.

### Viral cyclins and certain mammalian cyclins fully support endothelial cell survival and persistent infection

**Figure 3:**
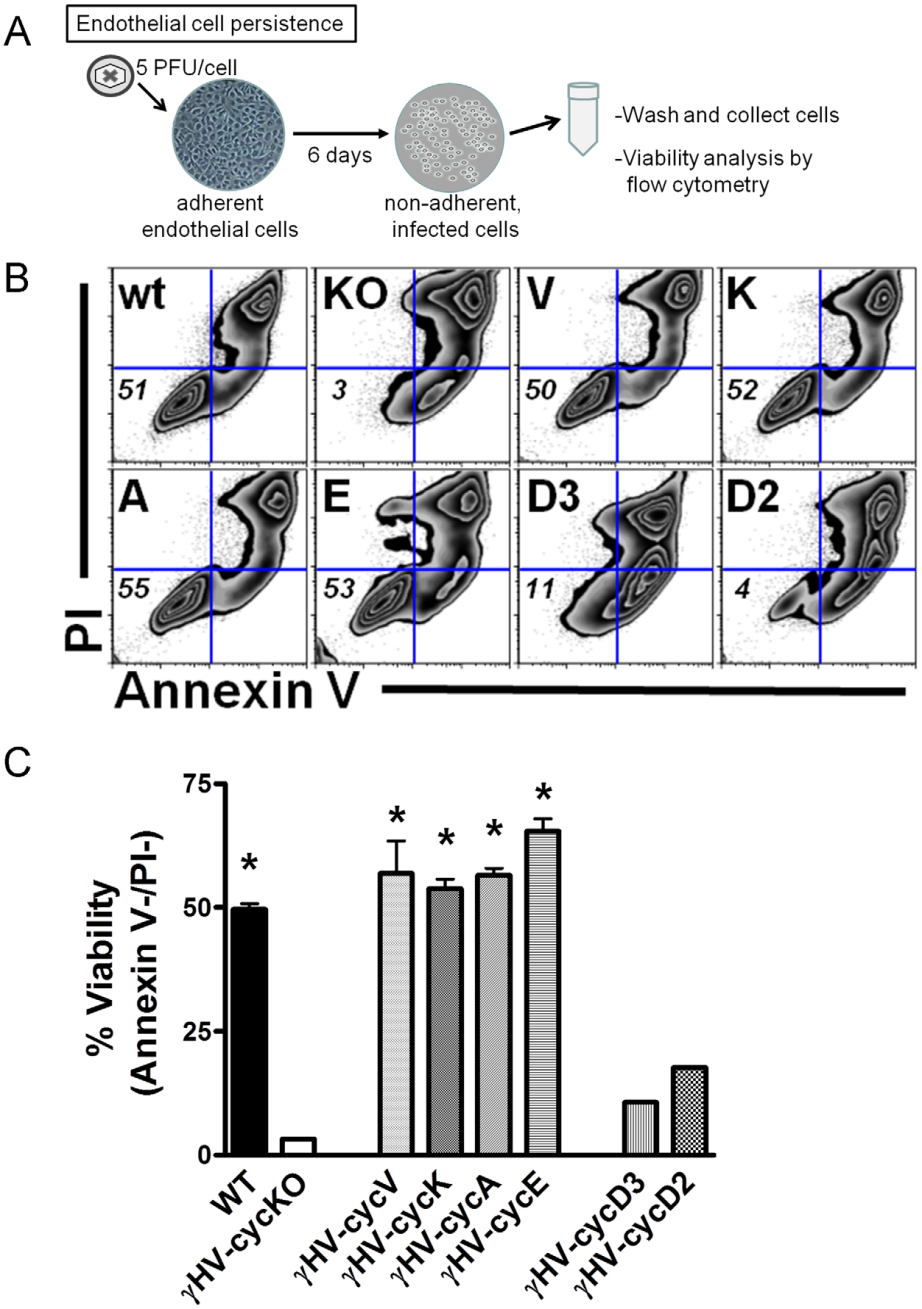
Both viral and mammalian cyclin-expressing viruses confer endothelial cell survival and persistence. (A) Schematic representation of persistent infection of endothelial cells for viability determination. (B) MB114 endothelial cells were infected with recombinant viruses for 6 days, at which time non-adherent cells were collected and analyzed for viability by dual PI and annexin V staining. Flow cytometry zebra plots are representative of 3 independent experiments from gHV-cycV, gHV-cycK, gHV-cycA, gHV-cycE, gHV-cycD3 and gHV-cycD2 virus infections. (C) Bar graph of mean viability (annexin V-/PI-) from flow cytometric analysis of 3 independent experiments. Data was found to be statistically significant between gHV-cycKO compared to WT (*p = 0.001), gHV-cycV (*p = 0.004), gHV-cycK (*p = 0.001), gHV-cycE (*p = 0.001) and gHV-cycA (*p = 0.001) as determined by unpaired t-test.

We previously reported that endothelial cells are able to support persistent gHV68 infection, a process that is dependent on the v-cyclin [20]. gHV68 infection results in a characteristic alteration in endothelial cell morphology, marked by modified gene expression and adherence-independent growth. These persistently infected endothelial cells remain viable for an extended time and are not lysed by virus infection, yet are productively infected and release abundant infectious virus. We next sought to determine the capacity of the recombinant cyclin viruses to promote survival and persistent infection in endothelial cells. Growth and survival of non-adherent surviving endothelial cells was measured at nine days post-infection (Figure 3A). Cell survival (percent annexin V- and PI-negative cells) following infection with the recombinant cyclin viruses is shown in Figure 3B. As in acute pulmonary infection, the KSHV k-cyclin and the gHV68 v-cyclin were both fully functional in endothelial cell persistent infection; however, no mammalian cyclins showed modest or intermediate capacities. Instead, mammalian cyclins A and E, which bear functional similarity to the viral cyclins, were fully functional (≥50% viability) in promoting persistent endothelial cell infection. In contrast, cyclins D3 and D2, which share the most sequence similarity to the viral cyclins, conferred no advantage over a cyclin deficient virus (Figure 3C). These data demonstrated that the viruses expressing D-type cyclins were completely defective in promoting endothelial cell survival (Figure 3C), equivalent to the defect observed with a virus completely deficient for the v-cyclin. In contrast, not only both viral cyclins, but also mammalian cyclins E and A, were able to fully restore endothelial cell persistence (Table 1) to the level conferred by the gHV68 v-cyclin.

**Table 1:** Cyclin-dependent parameters of infection and genetic complementation.

Parameters of infection	Compensating recombinant cyclin viruses
Endothelial cell survival/viral persistence	γHV-cycV, γHV-cycK, γHV-cycE, γHV-cycA
Reactivation from latency	γHV-cycV, γHV-cycK, γHV-cycD3
Acute virus production/pneumonia	γHV-cycV, γHV-cycK, γHV-cycD3**, γHV-cycE**, γHV-cycA**

**partial activity in given parameter.

### Cyclin function in reactivation from latency is restricted to the viral cyclins and mammalian cyclin D3

**Figure 4:**
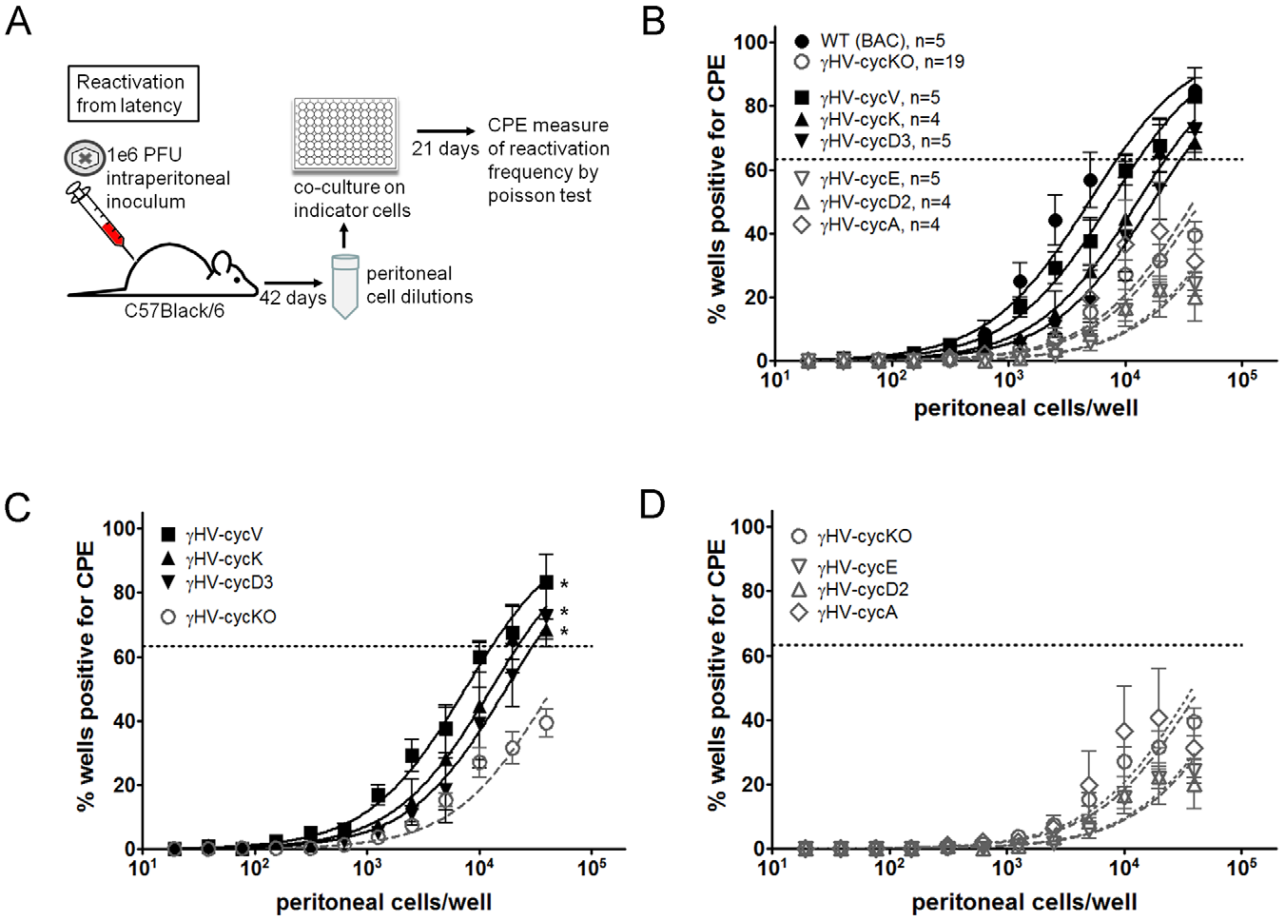
Viral cyclins and cyclin D3 compensate in reactivation from latency. (A) Schematic representation of infections and determination of ex vivo reactivation in C57BL/6 mice. (B–D) Limiting-dilution ex vivo reactivation from latency of PECs from mice infected with (B) compiled WT, gHV-cycKO and recombinant cyclin viruses, (C) gHV-cycV, gHV-cycK and gHV-cycD3 viruses that complement in reactivation and (D) gHV-cycA, gHV-cycE and gHV-cycD2 viruses that do not complement in reactivation. Data represent the mean ± SEM of the indicated number of independent experiments, with each experiment containing cells pooled from three to five mice and each experiment including the gHV-cycKO virus. The dashed line is at 63%, the value which was used to calculate the frequency of reactivating cells as indicated by a Poisson distribution. Reactivation frequencies were as follows: WT-gHV 1/8729, gHV-cycV 1/13152, gHV-cycK 1/19952, gHV-cycD3 1/25118; and less than 1/80000 for gHV-cycKO, gHV-cyc, gHV-cycE and gHV-cycA. Frequencies of reactivation were significantly increased by gHV-cycV (*p = 0.01), gHV-cycK (*p = 0.02) and gHV-cycD3 (*p = 0.03) compared to gHV-cycKO, as determined by paired t-test.

Latent infection with gammaherpesviruses is a complex process that is normally established in vivo, and is best measured after primary lytic infection has resolved. We previously used ex vivo analysis of cells infected with wild-type or cyclin deficient virus to show that the v-cyclin is critical to reactivation from latency [22] in both healthy and immune deficient mice [22], [25]. This requirement for the v-cyclin is surprising, given the presumed expression of the homologous host cyclins during infection. And while other viral genes are also required for reactivation from latent infection, to date, no other single gene has been found to play an equivalent role. To determine the required cyclin function in reactivation during infection of mice, we first established that the recombinant cyclin viruses behaved as expected in vivo; that is, no mortality was observed during six weeks of infection, the relative cellularity of splenic and peritoneal cells was consistent with WT infection at both 16 and 42 days post-infection (data not shown), and while infected cells are scarce, the FLAG-tagged v-cyclin can be detected in vivo (Figure S3A) during the peak of infection. Furthermore, we verified that reactivation of the FLAG-tagged recombinant virus was equivalent to that of the original WT virus (Figure S3B), and based on our previous demonstrations that the viral cyclin is not required for the establishment of latency [22], [25], we used a subset of these viruses (representing both those that do and do not complement reactivation from latency) to show that, as expected, latency was established normally (Figure S3C; no significant differences found in the frequency of latently infected cells). We previously demonstrated that the v-cyclin is required for both reactivation from latency and for persistent infection in immune deficient mice. Therefore we hypothesized that cyclin requirements for reactivation might be synonymous with those for persistence. This would predict complementation in reactivation by mammalian cyclins E and A, that is, that gHV-cycE and gHV-cycA would be significantly increased over gHV-cycKO. Infected peritoneal exudates cells (PECs) were plated on highly permissive MEF indicator cells for measurement of viral cytopathic effect (CPE) (Figure 4A). Reactivation analyses of the full panel of recombinant cyclin viruses are shown in Figure 4B, with cyclin recombinant viruses that restored v-cyclin function in reactivation shown in Figure 4C and those that fail to complement shown in Figure 4D. To our surprise, gHV-cycE and gHV-cycA failed to support reactivation, as did gHV-cycD2, with reactivation less than or equal to the cyclin deficient virus for each of these viruses (Figure 4D). Reactivation frequencies of the gHV-cycKO and the non-complementing viruses were from extrapolated values, as the cells reactivating fell short of 63% even at the highest concentration. We found that the viral cyclins of both gHV68 and of KSHV complemented v-cyclin function in reactivation, and that gHV-cycV and gHV-cycK infections resulted in reactivation frequencies that did not statistically differ from each other. In addition, gHV-cycD3 was the only mammalian cyclin virus that differed significantly from the gHV-cycKO virus in supporting reactivation from latency (Figure 4C). As expected from previous studies, the frequency of latently infected cells, or latency establishment, was similar between PECs infected with complementing versus non-complementing recombinant viruses (Figure S2D). These data demonstrate that reactivation from latency is supported by a cyclin activity common to the viral cyclins and mammalian cyclin D3 (Table 1), but that is not shared with mammalian cyclins E, A or D2.

## Discussion

Gammaherpesviruses establish lifelong infections in their host and are considered to be etiological agents for a variety of disease states, ranging from inflammatory conditions to malignancies, particularly in immunosuppressed individuals [34], [35]. While the precise mechanisms by which these viruses establish a chronic infection remains an ongoing area of investigation, one gene that clearly influences chronic infection is the viral cyclin, encoded by the human virus Kaposi's sarcoma associated herpesvirus and murine gammaherpesvirus 68. In KSHV, the k-cyclin is expressed during latency and reactivation from latency, and the k-cyclin regulates latency in KS cell lines [36], [37]. By using murine gHV68 infection of mice to assess the role of the v-cyclin in multiple stages of infection, we and others have found that the v-cyclin is necessary for multiple facets of chronic infection and pathogenesis, including acute virus production in the lung during immune deficiency [19], endothelial cell survival and viral persistence [20], and reactivation from latency [22]. Notably, the gHV68 v-cyclin is also required for chronic pathogenesis in immunosuppressed individuals, including the induction of chronic inflammatory conditions (e.g. in IFNgRKO; [25], [38]) and tumorigenesis (e.g. in BALB/b2M KO mice; [26]).

Given the varied roles that this gene has in promoting optimal gammaherpesvirus chronic infection and pathogenesis, there is a pressing need to understand the molecular mechanisms by which the v-cyclin mediates these diverse outcomes. While numerous reports have identified biochemical differences in the viral cyclins relative to host cyclins, to date there have been no studies to define which of these enhanced features of the viral cyclin are critical for gammaherpesvirus infection and pathogenesis. In fact, as reported here, analyses of kinase binding by viral and mammalian cyclins expressed under identical conditions during virus infection indicated multiple distinct patterns that do not correspond to subsequent functional studies. Additionally, kinase binding and activation may well differ in particular cell types and infection states in vivo, many of which are not readily amenable to biochemical analysis. Recently, mouse knock-outs and knock-ins have led to major advances in our understanding of cyclins and cdks, such that cyclins are now implicated not only in cell cycle progression, but in development, tissue specificity, tumorigenesis, and DNA damage and transcription, in the presence or absence of cdk partners. Using this successful approach, in this report, we tested the capacity of both viral and mammalian cyclins to function in multiple v-cyclin dependent stages of infection. Based on the enhanced biochemical features of the viral cyclins relative to mammalian cyclins, and the fact that host cyclins are present within the virus infected cell, we hypothesized that the viral cyclins of gHV68 and KSHV might be uniquely capable of functioning during virus infection. Indeed, when we first analyzed the ability of the various recombinant viruses to undergo replication in the lungs of immunosuppressed mice, we found that only the viral cyclins of either gHV68 or KSHV, and not mammalian cyclins, were capable of conferring wild-type levels of virus production and consequent increased pneumonia in infected lungs. These observations are consistent with the idea that the viral cyclins mediate their functions during infection through unique biochemical features, such as kinase binding, not present in mammalian cyclins. Notably, the viral cyclins of both gHV68 and KSHV were interchangeable in these tests of genetic complementation, despite this and previous reports identifying potential differences in their cdk binding partners and substrates [37], [39]–[41]. These data identify a genetically conserved mechanism of the gammaherpesvirus cyclins for in vivo infection and pathogenesis.

**Figure 5:**
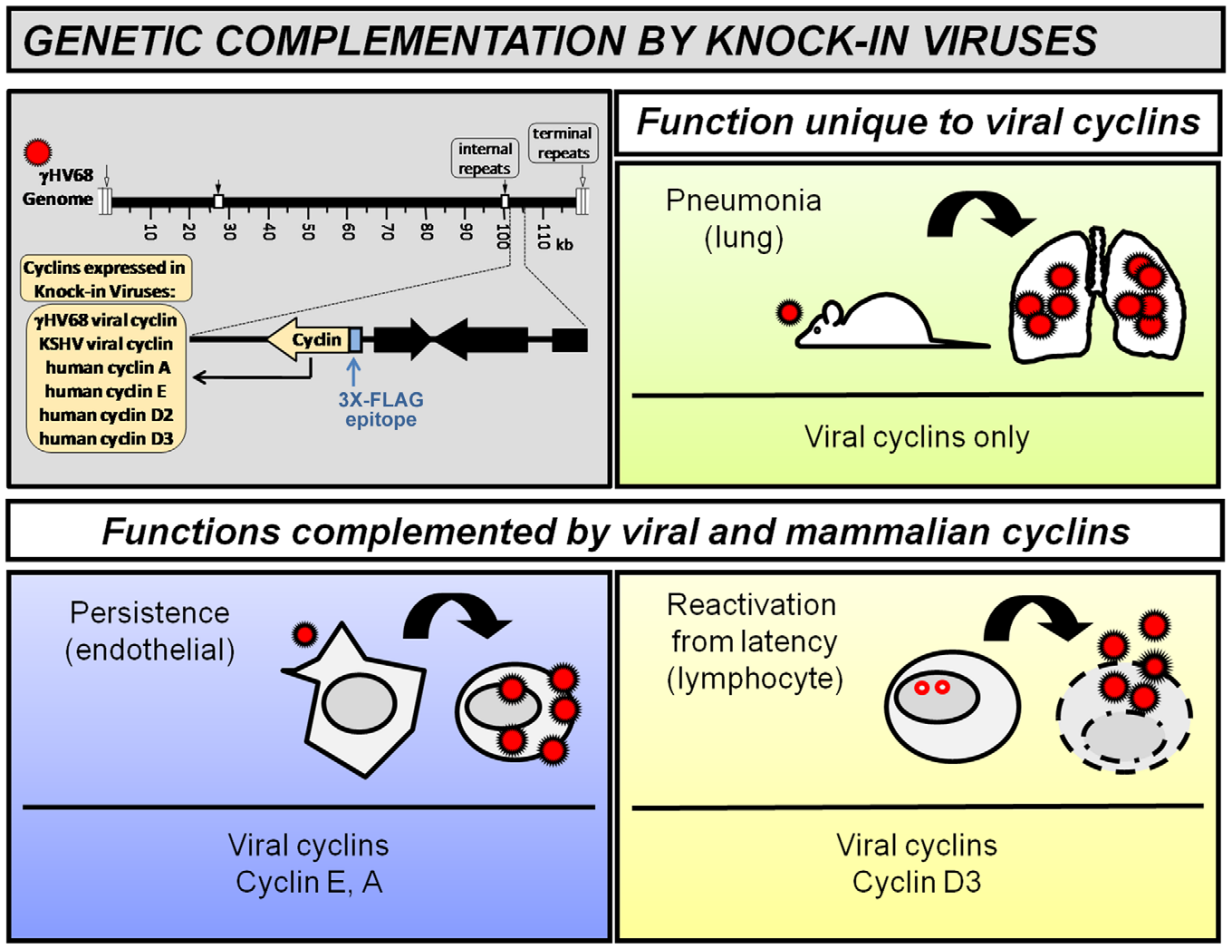
Proposed model for viral cyclin requirements in virus infection. Recombinant knock-in viruses with various viral or mammalian cyclins expressed from the endogenous gHV68 viral cyclin locus are depicted at upper left. Genetic complementation analysis of this panel of viruses demonstrated that the viral cyclins of gHV68 and of KSHV are functionally conserved and are unique in their ability to support all cyclin-dependent aspects of infection, depicted as three different panels. Further, mammalian cyclins able to function in virus infection comprised two genetically distinct groups, based on their ability to complement either persistent infection in endothelial cells (lower left) or reactivation from latency in lymphocytes (lower right). Finally, we propose that the unique capacity of the viral cyclins to support acute virus production and lethal pneumonia in the lungs of immune deficient mice does not reflect another distinct genetic requirement of viral cyclins. Rather, we hypothesize that both persistent infection and reactivation from latency contribute to optimal acute virus production in the lungs (upper right), as indicated by the combination of blue and yellow.

While only the viral cyclins provided optimal virus production in the immunosuppressed lung, further investigation of v-cyclin dependent stages of infection revealed a surprising ability of mammalian cyclins to mediate different stages of infection (model represented in Figure 5). On the one hand, host cyclins E and A was capable of fully promoting persistent infection of endothelial cells, while host cyclin D3 was capable of promoting reactivation from latency. Strikingly, the ability of host cyclins to mediate these distinct processes was non-overlapping, such that host cyclins capable of functioning in viral persistence were not capable of functioning in reactivation and vice versa. These data clearly demonstrate that, when expressed in the correct spatiotemporal manner (by insertion in the endogenous v-cyclin locus), host cyclins are able to mediate distinct subsets of v-cyclin dependent functions in vivo.

The existence of distinct genetic complementation groups of mammalian cyclins for optimal infection strongly suggests that these processes are mediated by distinct molecular mechanisms. Based on the ability of host cyclins E and A, but not D-type cyclins, to promote viral persistence it is worth asking what this complementation pattern might tell us about how the v-cyclin promotes persistence. What unique features do cyclins E and A have that differ from the D-type cyclins? First, cyclins E and A (and the v-cyclins) differ in kinase partners, but all confer stronger kinase activation and longer half-lives than the D-type cyclins. While cyclins E and A may function by promoting cell cycle progression, herpesvirus infection is also associated with cell cycle arrest [42]. A second possible explanation for cyclins E and A in promoting endothelial cell persistence might be the fact that a cellular DNA damage response is important in promoting early herpesvirus DNA replication [43], and these cyclins are important in the DNA damage response [44], induction of which correlates to strength of kinase activation [45]. It is also worth noting that viral persistence is dependent on host autophagy machinery and an ability to survive substrate detachment as well [46]. These studies clearly demonstrate that the requirement for cyclin function in endothelial cell persistent infection corresponds to capacity for kinase activation. Based on this, we hypothesize that viral persistence may be particularly sensitive to therapeutic kinase inhibitors.

Reactivation of virus replication from latently infected cells is a critical function of the v-cyclin in vivo, and correlates well with pathologies of chronic infection [25], [26], [38]. Because latent infection over time is concentrated in quiescent memory B cells, one straightforward possibility is that the v-cyclin is required in reactivation simply to stimulate quiescent cells into cycle. However, past reports have indicated that induction of the cell cycle by immunoglobulin cross linking or Toll-like receptor stimulation is insufficient to overcome the defect in reactivation that is observed with the v-cyclin-deficient virus [21], [47]. If cell cycle progression via cdk activation is the sole requirement for reactivation, then redundancy in cell cycle function [14] predicts that proper expression of any cyclin should substitute for v-cyclin in reactivation. Instead we found that only the viral k-cyclin and mammalian cyclin D3 were able to genetically function in promoting reactivation from latency. These data compellingly indicate that reactivation from latency is dependent on a highly restricted cyclin activity possessed by cyclin D3 that does not correspond to kinase requirement in cell cycle progression, in which cyclins E and A can substitute for D-type cyclins and cyclin D/cdk4 or cdk6 complexes are not required for cell cycle [16]. This observation is consistent with the demonstration of wild-type reactivation following infection with mutant v-cyclin viruses that are impaired in cdk binding in vitro [48], and with reports of cdk-independent functions of D-type cyclins [49]–[51]. The inability of cyclin D2 to compensate in reactivation is not likely a feature of poor protein stability [52], because this is a shared feature of the D-type cyclins. Instead, our data suggest that the ability of mammalian cyclin D3 to function in reactivation is based on a unique role for cyclin D3, such as transcription regulation [53]–[55], activation of unconventional kinase partners [56], [57], or cell type-specific function. It is worth noting that a unique role for cyclin D3 in promoting virus infection has also been observed in promoting herpes simplex virus reactivation [58]. Given that B lymphocytes are the major latency reservoir of the gammaherpesviruses, it is notable that cyclin D3 is specifically required in lymphocyte development [59], and particularly in germinal center B cells, a prominent early reservoir for viral latency [60], [61].

Beyond specific insights into the mechanisms by which the v-cyclin promotes chronic infection, this study also revealed a fundamental new insight in gammaherpesvirus infection, by demonstrating that viral reactivation from latency and viral persistence are genetically separate processes. To date, these processes are frequently intertwined spatially and temporally, making it difficult to discern their interrelationship. These distinct cyclin functions suggest a new explanation for the partial complementation of the mammalian cyclins during the acute phase of replication in the lung. While the general assumption that primary lytic virus replication is cleared and then followed by latent infection, Flano, et al. provided evidence that lytic and latent infection occur simultaneously and early in the lungs, and that latently infected cells are apparent as early as three days post-infection [62]. Our study further supports this finding, and provides genetic evidence that reactivation from latency, generally considered only in later stages of infection, contributes to virus production during the early stages of primary infection. Previously, persistent infection (as defined by detection of infectious virus late in infection) and reactivation were both increased in immune-deficient mice, consistent with increased reactivation from latency resulting in increased persistent infection, or vice versa [38]. Two viral homologs of host genes, the viral cyclin and the viral bcl-2, are both required in persistence and in reactivation. The first indication that these are separable processes was identified by recent analysis of the viral bcl-2 homolog, in which different v-bcl-2 mutants were capable of supporting either persistence or reactivation [63]. Further, we demonstrated that both the v-cyclin and the v-bcl-2 are required for optimal virus production and lethal pneumonia in immune-deficient hosts [19]. First, it is remarkable that the genetic requirements for both the v-cyclin and the v-bcl-2 are separable in these distinct aspects of virus infection. Second, these data illustrate that in immune-deficient mice, acute virus production cannot be solely attributed to primary virus replication, but may be the sum of replication, persistence, and reactivation. Genetic separation of these functions raises the potential that chronic disease previously associated with both persistence and reactivation may be dependent on one or the other. The cyclin recombinant viruses now provide a mechanism to determine the relative contribution of reactivation and persistence in various disease processes, and may provide insight for therapeutic interventions specifically tailored to the cyclin susceptibility of each.

In total, our findings demonstrate that the multifunctional nature of the viral cyclins described in *in vitro* biochemical studies corresponds to genetically distinct and required functions during virus infection, and that both the gHV68 and KSHV viral cyclins share this multifunctional capacity in infection. Additionally, this study revealed distinct genetic complementation groups of the mammalian cyclins, demonstrating that mammalian cyclins can fulfill the biochemical features of the v-cyclin in infection. These studies reveal that the unusual biochemical features of viral cyclins, such as broad substrate specificity and increased kinase activity, are not absolutely required to mediate specific processes within viral infection. And yet, cyclin D3 restored v-cyclin dependent reactivation less effectively than did the viral cyclins, suggesting that unique biochemical feature(s) of viral cyclins may be required to facilitate robust activity in reactivation. These data also indicate that v-cyclin features, such as resistance to cell cycle inhibitors or enhanced kinase activity, are necessary for optimal gammaherpesvirus pathogenesis.

The unique capacity of the viral cyclins to encompass functions of multiple mammalian cyclins probably explains the evolutionary advantage of encoding viral cyclins within the viral genome. Whereas only the viral cyclins can perform all v-cyclin dependent parameters of infection, our data also suggest that expression of endogenous host cyclins could complement v-cyclin-dependent functions in vivo. This idea is consistent with our observations that neither persistent infection nor reactivation from latency is completely abrogated in absence of the v-cyclin. Since mammalian cyclins can genetically replace the v-cyclin in distinct stages of infection, we hypothesize that methods of interfering with mammalian cyclin-mediated processes may also be effective at inhibiting specific functions of the v-cyclin. The ultimate test of this idea will be specific chemical inhibition of specific v-cyclin functions, and whether such inhibition indeed decreases persistent infection and reactivation levels below that of v-cyclin deficient viruses. Finally, the distinct cyclin requirements in different v-cyclin stages of infection provide potential for specific treatment of different gammaherpesvirus pathologies using existing therapeutic inhibitors specific to certain host cyclins and cdks [64]. Because cyclins and cdks are well-conserved and are host proteins, this strategy circumvents potential virus escape and may also prove useful for treatment of herpesviruses that do not encode cyclins within their genomes.

## Materials and Methods

### Ethics statement

This study was carried out in strict accordance with the recommendations in the Guide for the Care and Use of Laboratory Animals of the National Institutes of Health. All animal studies were conducted in accordance with the University of Colorado Denver Institutional Animal Use and Care Committee under the Animal Welfare Assurance of Compliance # A3269-01. All surgery was performed under isoflurane anesthesia, and all efforts were made to minimize suffering.

### Viruses, cell lines and tissue culture

gHV68 clone WUMS (WT; ATCC VR1465), gHV68 containing a stop codon within ORF 72 (cycKO), and parental and epitope-tagged recombinant viruses (Figure S1) were passaged and grown, and the titer was determined as previously described [22], [31]. NIH 3T12 (ATCC CCL-164), Vero-Cre cells (Dr. David Leib, Dartmouth Medical School of Medicine) and mouse endothelial cell lines MB114 [65] were grown in Dulbecco's modified Eagle medium (DMEM) supplemented with 5% fetal bovine serum (FBS), 100 U/ml penicillin, 10 ug/ml streptomycin sulfate, and 2 mM L-glutamine. Mouse embryonic fibroblasts (MEFs) were isolated from C57/BL6 mice as previously described [66] and cultured in DMEM supplemented with 10% FBS, 2 mM L-glutamine, 10 U/mL penicillin, 10 µg/mL streptomycin sulfate, and 250 ng/mL amphotericin B. Infection of MB114 endothelial cells was carried out at a multiplicity of infection (MOI) of 5 plaque forming units (PFU) per cell, as previously described [20]. The inoculum was removed after one hour of infection at 37°C, and the cell monolayers were cultured in complete media after rinsing with PBS. Intact and non-adherent cells were collected at six days post-infection, at which time cells and media were collected [20].

### Mice, infections, and organ harvests

C57BL/6 and IFN-g^−/−^ mice on a BALB/c background (strain C.129S7(B6)-*Ifng^tm1Ts^*/J) were purchased from The Jackson Laboratory (Bar Harbor, ME). Eight to ten week old mice were infected by intraperitoneally (i.p.) with 1×10^6^ PFU of virus in 0.5 ml of DMEM/5% FBS for reactivation studies and intranasally (i.n.) with 4×10^5^ PFU of virus in 40 µl of DMEM/5% FMS for acute infection studies. Upon sacrifice, lungs for which virus titers were to be determined were placed in 1 ml of DMEM/5% FBS on ice and frozen at 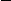
80°C [19]. Peritoneal cells (PECs) were harvested by peritoneal lavage with 10 ml of DMEM/1% FBS [22].

### Southern blotting

Viral DNA was generated by infection of 3T12 cells at an MOI of 0.05 for each recombinant virus. Infected-cell cultures were harvested at 50% CPE, and DNA was prepared as previously described [31]. 5–10 ug of viral DNA was restriction enzyme-digested for four hours. Digests were electrophoresed on 0.8% agarose gels with biotinylated DNA ladders (New England Biolabs, Ipswich, MA). The DNA was alkaline transferred to Zeta-Probe membrane (Bio-Rad Laboratories, Hercules, CA) using the Turboblot apparatus (Schliecher & Schuell, Keene, NH), according to the manufacturer's recommendations. Probes were from gHV68 genome coordinates 101656 to 105385 (cyclin region probe) or 11100 to 16328 region (left end probe). Each probe was biotinylated and quantitated, according to manufacturer's instructions for the KPL Detector HRP chemiluminescent blotting kit (KPL, Inc., Gaithersburg, MD).

### Viruses, plaque assays and determination of viral titers

gHV68 clone WUMS (ATCC VR-1465) and recombinant viruses were passaged and grown, and titer determined as previously described [31]. Plaque assays were performed on 3T12 cells as previously described [19], [25]. Lung homogenates were serially diluted, and plated onto NIH 3T12 cells in 12 well plates in triplicate. The limit of detection of the assay is 50 PFU. Viral replication in vitro was determined by infection of 3T12 cells at a MOI of 0.05 PFU per cell to measure multiple cycle replication. Cells and supernatants were collected at various times post-infection and frozen at 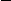
80°C. Samples were subjected to four cycles of freezing and thawing prior to quantitation by plaque assay.

### Transfections

Plasmids and BAC DNA were introduced into cells using the calcium phosphate method. 293T, Vero-Cre or 3T12 cells were plated in 6-well plates and transfected at 50–80% confluency. The DNA mixture for each well, which consisted of the 1–10 ug DNA, 2 M CaCl_2_, and sterile H_2_0, was combined with 2× HEPES balanced saline buffer (0.3 M NaCl, 0.05 M HEPES, 0.003 M Na_2_HP0_4_, H_2_0; pH 7.05–7.15) and added to each well dropwise while gently swirling plates. At 16 hours post transfection, cells were washed with 1× phosphate buffered saline, the media were replaced with DMEM/5% FBS, and cells were examined by fluorescence microscopy at various times post-transfection to monitor transfection efficiency or BAC-GFP deletion.

### Quantitative reverse transcription PCR

Total RNA was isolated from infected 3T12 cells using TRIzol Reagent (Invitrogen) and then purified using the RNeasy Micro Kit (Qiagen). An ABI Prism 7900 sequence detector (Applied Biosystems, Foster City, CA, USA) was used for measurement of the fluorescence spectra in a thermal cycler during PCR amplification (University of Colorado Cancer Center Quantitative PCR Core Facility). Forward and reverse primers and probe (Applied Biosystems) specific to the 3x-FLAG-CMV 7.1 epitope were designed according to the recommendations of the TaqMan PCR chemistry design and optimized using the Primer Express software (Applied Biosystems). Primer and probe sequences used were 3x-FLAG7.1FWD-CTACAAAGACCATGACGGTGATTATAA; 3x-FLAG7.1REV.NEW-TCGCGGCCGCAAGC; 3x-FLAG7.1PROBE-6-carboxyfluorescein-CATGACATCGATTACAAGGATGACGATGAC-6-carboxy-tetramethylrhodamine. Amplification reactions and thermal cycling conditions were performed as per the manufacturer's recommendations. A standard curve was created using the fluorescence data from 10-fold serial dilutions of a 24 hour 3x-FLAG-v-cyclin infection. The 24 hour 3x-FLAG data were normalized to the 12 hour 3x-FLAG data, and is represented as the ratio of 3x-FLAG RNA to the total amount of 18S rRNA per sample.

### Antibodies, immunoprecipitations, immunoblotting and immunohistochemistry

The following antibodies were used: mouse anti-Flag (M2, Sigma-Aldrich), rabbit anti-Flag (Cell Signaling Technology, Inc, Danvers, MA), rabbit anti-v-cyclin[11]), rabbit anti-k-cyclin [gift from Sibylle Mittnacht [67], rabbit anti-cdk1/cdc2, goat-anti-cdk2, goat anti-cdk4, rabbit anti-cdk6, rabbit-anti-cyclin A, rabbit anti-cyclin D3, rabbit anti-cyclin E (Santa Cruz Biotechnology, Inc., Santa Cruz, CA), mouse anti-beta-actin (Sigma), and donkey anti-rabbit-HRP, donkey anti-mouse-HRP, donkey anti-goat-HRP (Jackson ImmunoResearch Laboratories, Inc, West Grove, PA). Protein expression was detected by lysing cells in ELB buffer (50 mM HEPES pH 7.2, 250 mM NaCl, 2 mM EDTA, 0.1% NP-40) for 20 minutes on ice, and boiling for 10 minutes. Equal cell equivalents or equal amounts of protein, based on RC-DC protein assay (Bio-Rad) were loaded per lane. Samples were separated by electrophoresis on 7.5%–15% denaturing polyacrylamide gels and transferred to Immobilon-P membranes (Millipore Corp., Bedford, MA) by semi dry protein transfer (Panther Semi Dry Electroblotter, Thermo Fisher Scientific, Inc., Portsmouth, NH), and analyzed ECL Plus western blotting detection reagents (GE Healthcare, Piscataway, NJ). 10% of each cell lysate was set aside for lysate loading controls in immunoprecipitations. Remaining lysates in ELB containing protease inhibitors (1 mM DTT, 10 mM NaF, 50 ug/mL PMSF, 1 ug/mL aprotinin, 1 ug/mL leupeptin) were precleared with protein A sepharose CL-4B beads (GE Healthcare) for one hour at 4°C with agitation. Lysates were then clarifed, and incubated for one hour at 4°C with anti-Flag Ab (Sigma) prior to the addition of sepharose beads and overnight incubation. Beads were washed four times in cold ELB with inhibitors, boiled for 10 minutes in Laemmli buffer (0.25 M Tris-HCl pH 6.8, 2% SDS, 10% glycerol, 5% b-mercaptoethanol, 0.002% bromophenol blue) and subjected to SDS-PAGE. Cells infected for immunohistochemical detection of 3x-FLAG cyclins were fixed using 3∶1 methanol: glacial acetic acid. 20 ug/ml of mouse anti-FLAG (Sigma) was added to the cover slips and visualized with goat anti-mouse Alexa Fluor 568 (1∶1000; Invitrogen). For ex vivo FLAG detection, four-six µm sections were deparaffinized and before antigen retrieval using 10 mM citrate buffer. Tissues were blocked using 10% 2.4G2 and 5% goat serum in PBS prior to staining with rabbit anti-FLAG at 1∶500 (Cell Signaling) followed by biotin goat anti-rabbit at 1∶50 (BD Pharmingen) and streptavidin-RPE (Invitrogen) at 1∶100. All slides were mounted with ProLong Gold antifade reagent with 4′-6-diamidino-2-phenylindole (DAPI, Invitrogen) and images were obtained using an Olympus IX81 inverted motorized scope with spinning disk (Olympus, Center Valley, PA), a Hamamatsu ORCA IIER monochromatic CCD camera (Hamamatsu, Bridgewater, NJ) and Intelligent Imaging Slidebook v.4.067 (Intelligent Imaging Innovations, Denver, CO).

### Limiting-dilution genome analysis

The frequency of cells containing viral DNA was determined by a limiting-dilution nested-PCR assay that amplifies gHV68 gene 50 sequences with approximately single-copy sensitivity, as described previously [22], [68]. Briefly, peritoneal cells (PECs) were harvested from latently infected mice and plated as a limiting-dilution series of cells. The cells were lysed prior to PCR amplification, and the first-round PCR product served as a template for the second round of PCR amplification. Control reactions of uninfected cells (negative control) or plasmid DNA (pBamHIN) of known copy number (positive control) were included in each experiment [25].

### Limiting-dilution ex vivo reactivation assay

Quantitation of gHV68 reactivation from latency was performed as previously described [22], [68], [69]. Briefly, PECs were harvested from infected mice at day 42–50 post-infection, and single-cell suspensions were generated. Two-fold serial dilutions of infected cells were plated onto MEFs and scored for CPE after 21 days of co-culture. To detect preformed infectious virus, parallel samples were mechanically disrupted as previously described [25].

### Flow cytometry

Two parameter viability studies using propidium iodine (PI) and annexin V were performed as previously described [20] and analyzed by FlowJo (Treestar, Ashland,OR).

### Histology

For histologic examination, lungs were fixed in 10% formalin, paraffin embedded, sectioned (4–6 µm) and stained with H&E for analysis using a Zeiss Axiocam HR camera and KS 300 Imaging System 3.0 software [27]. Pulmonary disease was evaluated by board certified pathologist, Dr. Carlyne Cool.

### Statistical methods

All data was analyzed by using GraphPad Prism software (GraphPad Software, San Diego, CA). Viral titers were statistically analyzed with a one-way ANOVA test. Differences in endothelial cell survival were statistically analyzed by unpaired *t*-test. The frequencies of reactivation and genome-positive cells were statistically analyzed by paired *t*-test. Frequencies of latently infected and reactivating cells were obtained from the cell number at which 63% of the wells scored positive for either reactivating virus or the presence of the viral genome based on the Poisson distribution. Data were subjected to nonlinear-regression analysis to obtain the single-cell frequency for each limiting-dilution analysis.

### Accession numbers

Genbank accession numbers for proteins studied within this manuscript: gHV68 cyclin AAB66456; KSHV cyclin ADQ57958; human cyclin A2 AAM54042; cyclin E1 AAH35498; cyclin D3 AAA52137; cyclin D2 AAH89384.

## Supporting Information

Figure S1 **Generation and verification of recombinant cyclin viruses.** We used the gHV68 model system to generate an extensive panel of recombinant viruses in which the v-cyclin was uniformly and precisely replaced with the original gHV68 v-cyclin, the KSHV viral cyclin (k-cyclin), or the mammalian cyclins D2, D3, E and A. Each cyclin in this panel was tagged with a 3x-FLAG epitope to provide identical and sensitive detection. (A) Schematic representation of p3x-FLAG plasmids for epitope-tagged cyclin expression. Each cyclin cDNA was PCR amplified with primers listed in Table S1 to facilitate directed cohesive end cloning between NotI and BamHI/BglII sites. p3x-FLAG and cyclin cDNA sequences with restriction endonuclease cloning sites are indicated, along with initiation and termination codons and dotted line represents plasmid backbone sequence. The resulting 3x-FLAG-cyclin coding sequence of each plasmid was sequence verified. (B, C) 3x-FLAG-cyclin expression from these plasmids was determined by transfection of 2 ug of each plasmid into 293T cells with pMaxGFP control plasmid to monitor transfection efficiency. Cells were harvested 48 hrs later, lysates resolved on a polyacrylamide gel, transferred and probed with (B) cyclin- or (C) FLAG-specific antibodies. Each plasmid expressed a 3x-FLAG-cyclin fusion protein at the expected size that was uniformly detected by the FLAG antibody and specifically detected by antibodies to each cyclin. (D) Schematic representation of pGS-3700 targeting plasmid containing a 3723 bp fragment viral genomic sequence including the viral cyclin from positions 101,654 and 105,377 (shown in reverse orientation here) in the pGS284 BAC recombination vector, and modifications to create the pGS3700. cycFdr (founder) plasmid for uniform insertion of cyclin coding sequences. Quikchange mutagenesis was performed using primers listed in Table S1 to make the following modifications: 1) an *AvrII* site introduced at the terminal TAG, 2) inactivation of the endogenous polyA site (internal to the open reading frame) with a silent mutation, and 3) insertion of an identical polyA site directly following the terminal TAG, 18 bp from the original polyA site. Targeting plasmids for all recombinant cyclin viruses were generated by insertion of the cyclin cDNAs from p3X-FLAG plasmids between the *NcoI* and *AvrII/NheI* sites of the pGS-3700.cycFdR targeting plasmid. A *NcoI* to *NsiI* fragment of pL3700.stop [22] was inserted into the corresponding region in the v-cyclin gene of pGS-3700.cycFdR, to create pGS-3700.cycFdR.stop, containing a translational stop linker, and referred to throughout text as gHV-cycKO. The gHV68 cyclin and M11 coding sequences are indicated, and gHV68 genomic sequence is indicated by dashed line. Initiating methionine, termination codon, poly A sites, and relevant restriction enzyme sites are indicated. A gHV68 BAC containing a kanamycin gene (kanamycin resistance cassette was PCR amplified from pCR-TOPO-Kan (Invitrogen), inserted at bp160 of the v-cyclin gene in pGS-3700 to create pGS-3700-Kan; primers listed in Table S1) was generated via bacterial recombination with the parental γHV68 BAC [70] and selected via kanamycin and SacB resistance using methods previously described [71], [72]. 3x-FLAG cyclin targeting plasmids were then recombined with the KanR BAC to generate epitope tagged cyclin recombinant viruses. Potential recombinant clones were each screened by PCR, restriction mapping, and Southern blotting prior to final sequence verification. Finally, all BAC recombinants for cyclin analysis were transfected into Vero-Cre cells for removal of BAC sequences prior to infection of 3T12 fibroblasts for generation of virus stocks. (E, F) Representative Southern analysis of BAC-derived recombinant viruses. (E) Purified genomic viral DNA was digested with *Pst*I (left) or *Eco*RI (right), electrophoresed, blotted, and hybridized with the cyclin region probe. (F) To assess the integrity of the left end of the viral genome, purified viral DNA was digested with *Not*I and *Nsi*I, electrophoresed, blotted, and probed with a left end fragment of the viral genome. (G) Single step viral replication. 3T12 cells were infected with cyclin recombinant viruses at a MOI of 5.0 PFU/cell and harvested at various times as indicated. We found similar viral titers among all of the original gHV68 viruses and the parental viruses of the cyclin recombinants. Thus, replacement of the v-cyclin of gHV68 with other cyclin genes does not alter virus replication in vitro.
(TIF)

Figure S2 **Cyclin recombinant viruses express 3x-FLAG cyclins and associate with cellular cdks during infection in vitro.** Cells were infected with media (mock), WT or 3X-FLAG cyclin viruses at a MOI of 5 PFU/cell and harvested at 24 or 48 hours post-infection. (A) Recombinant cyclin viruses express each cyclin protein abundantly during lytic infection of MB114 endothelial cells. Lysates from cells infected for 24 hours were transferred and probed with antibodies to beta-actin, followed by the Flag epitope. 10 µg of lysate was loaded per lane and data is representative of three independent infection and immunoprecipitation experiments. (B) Lysates from 3T12 fibroblasts or MB114 endothelial cells were harvested at 48 hours post-infection and immunoprecipitated with anti-Flag antibody prior to resolution of lysates and immunoprecipitations on a 12% polyacrylamide gel. After transfer, blots were probed with antibodies to cdks indicated at right. Total lysate samples are on the left and Flag-IP samples on the right, as indicated for each blot. Treatments and infections are indicated above each lane. The IP immunoblots shown.are representative of three independent infections and immunoprecipitations performed in each cell type. Note that cdk band intensities of gHV-cycV and gHV-cycK IPs are dramatically different from those of the mammalian cyclin expressing viruses, which, though consistent, are faint by comparison. (C) Viral cyclin localization is similar between gHV68 and KSHV viral cyclins during lytic infection. 3T12s were infected with WT, gHV-cycV and gHV-cycK viruses at an MOI of 5 PFU/cell and harvested at 12 and 24 hours post-infection. Cells were stained with anti-FLAG antibody and detected with Alexa Fluor 568 secondary antibody (red) and DAPI (blue). Original magnifications ×40. Representative images are shown from two independent experiments.
(TIF)

Figure S3 **Cyclin recombinant viruses express 3x-FLAG cyclins and behave as expected during infection in vivo.** (A) Viral cyclins are expressed during infection in vivo. Lung sections from C57BL/6 mice infected for 16 days with WT or gHV-cycV viruses were stained with anti-FLAG antibody and detected with Alexa Fluor 568 secondary antibody (red) and DAPI (blue). White arrows indicate positive FLAG staining, original magnifications ×40. Specific anti-Flag signal was detected in 3X-V-infected lungs at day 16 post-infection, with six Flag-positive cells counted in 12 fields of gHV-cycV infected lung examined, indicating approximately one cyclin-positive cell per 372 lung cells during establishment of latency. This is the first report of direct detection of the v-cyclin protein in infected tissue. (B,C) Limiting dilution analysis of latency (cyclin-independent) and reactivation (cyclin-dependent) of cyclin recombinant viruses following infection of C57BL/6 mice. Data represent the mean ± SEM of two-four independent experiments. (B) Frequency of spleen cells reactivating virus replication (wells resulting in cytopathic effect) from latency at 16 dpi. (C) Frequency of latently infected peritoneal cells (viral genome-positive cells) at 42 dpi. Frequency of latently infected cells was not statistically different among WT, gHV-cycKO, gHV-cycD3, gHV-cycK and gHV-cycD2. The dashed line at 63% indicates the value that was used to calculate frequency based on the Poisson distribution.
(TIF)

Table S1 **Oligonucleotide sequences used in generation and analysis of plasmids and viruses.**
(TIF)
