# Supplementary figures and images for: Viral Cyclins Mediate Separate Phases of Infection by Integrating Functions of Distinct Mammalian Cyclins
Source: PLoS Pathog. 2012 Feb 2;8(2):e1002496. doi: 10.1371/journal.ppat.1002496 (PMC3271081; doi:10.1371/journal.ppat.1002496)

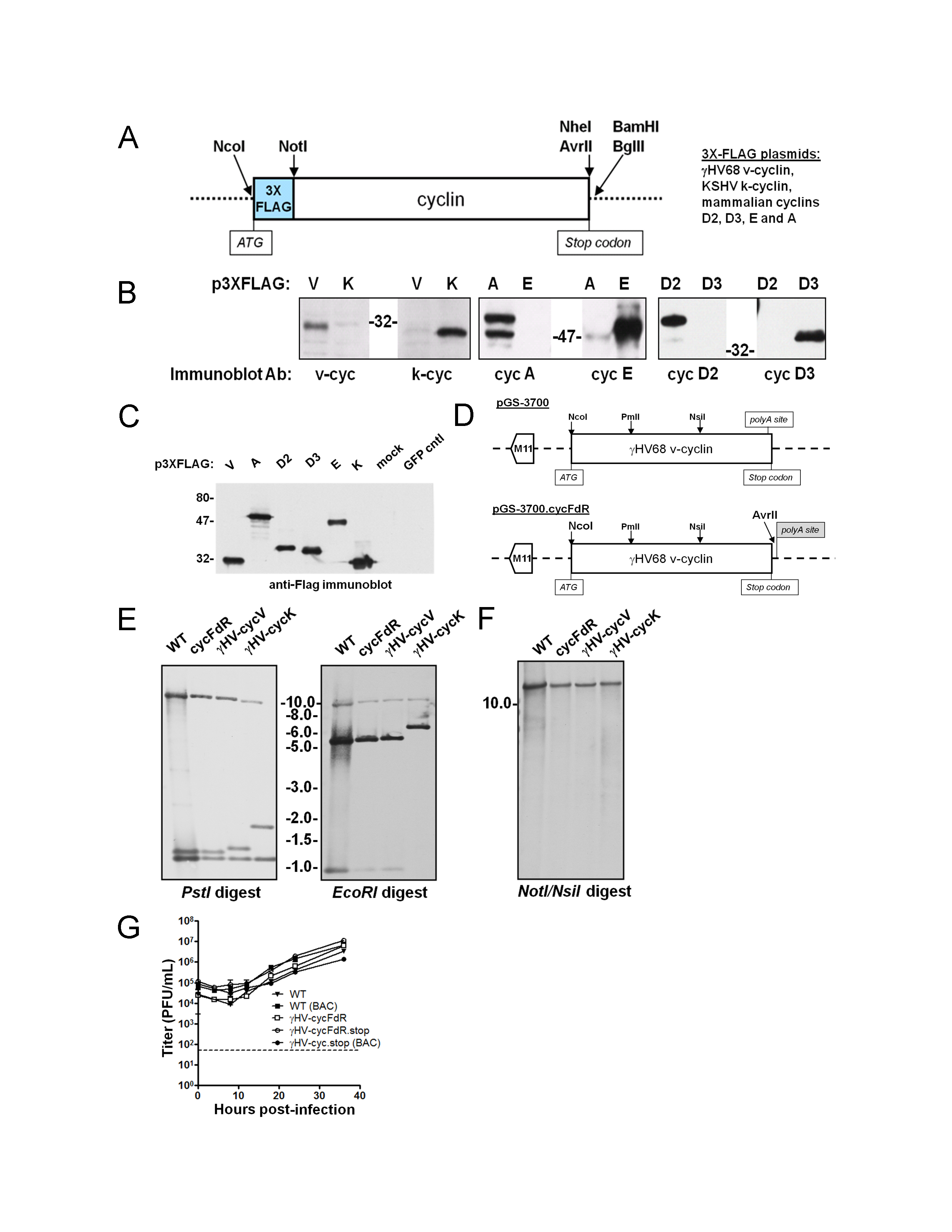

Supplement: Figure S1 — Generation and verification of recombinant cyclin viruses. We used the gHV68 model system to generate an extensive panel of recombinant viruses in which the v-cyclin was uniformly and precisely replaced with the original gHV68 v-cyclin, the KSHV viral cyclin (k-cyclin), or the mammalian cyclins D2, D3, E and A. Each cyclin in this panel was tagged with a 3x-FLAG epitope to provide identical and sensitive detection. (A) Schematic representation of p3x-FLAG plasmids for epitope-tagged cyclin expression. Each cyclin cDNA was PCR amplified with primers listed in Table S1 to facilitate directed cohesive end cloning between NotI and BamHI/BglII sites. p3x-FLAG and cyclin cDNA sequences with restriction endonuclease cloning sites are indicated, along with initiation and termination codons and dotted line represents plasmid backbone sequence. The resulting 3x-FLAG-cyclin coding sequence of each plasmid was sequence verified. (B, C) 3x-FLAG-cyclin expression from these plasmids was determined by transfection of 2 ug of each plasmid into 293T cells with pMaxGFP control plasmid to monitor transfection efficiency. Cells were harvested 48 hrs later, lysates resolved on a polyacrylamide gel, transferred and probed with (B) cyclin- or (C) FLAG-specific antibodies. Each plasmid expressed a 3x-FLAG-cyclin fusion protein at the expected size that was uniformly detected by the FLAG antibody and specifically detected by antibodies to each cyclin. (D) Schematic representation of pGS-3700 targeting plasmid containing a 3723 bp fragment viral genomic sequence including the viral cyclin from positions 101,654 and 105,377 (shown in reverse orientation here) in the pGS284 BAC recombination vector, and modifications to create the pGS3700. cycFdr (founder) plasmid for uniform insertion of cyclin coding sequences. Quikchange mutagenesis was performed using primers listed in Table S1 to make the following modifications: 1) an AvrII site introduced at the terminal TAG, 2) inactivation of the endo [file ppat.1002496.s001.tif]

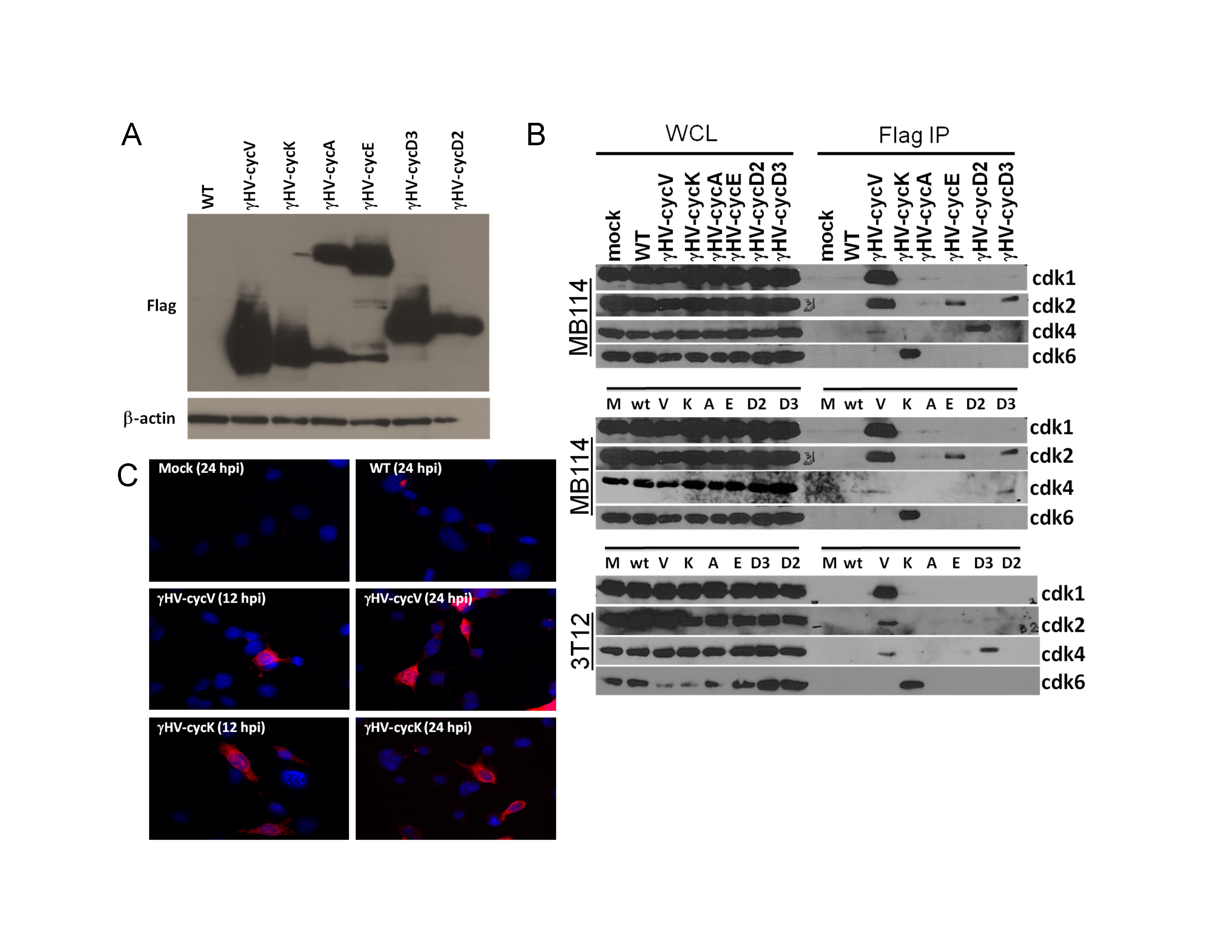

Supplement: Figure S2 — Cyclin recombinant viruses express 3x-FLAG cyclins and associate with cellular cdks during infection in vitro. Cells were infected with media (mock), WT or 3X-FLAG cyclin viruses at a MOI of 5 PFU/cell and harvested at 24 or 48 hours post-infection. (A) Recombinant cyclin viruses express each cyclin protein abundantly during lytic infection of MB114 endothelial cells. Lysates from cells infected for 24 hours were transferred and probed with antibodies to beta-actin, followed by the Flag epitope. 10 µg of lysate was loaded per lane and data is representative of three independent infection and immunoprecipitation experiments. (B) Lysates from 3T12 fibroblasts or MB114 endothelial cells were harvested at 48 hours post-infection and immunoprecipitated with anti-Flag antibody prior to resolution of lysates and immunoprecipitations on a 12% polyacrylamide gel. After transfer, blots were probed with antibodies to cdks indicated at right. Total lysate samples are on the left and Flag-IP samples on the right, as indicated for each blot. Treatments and infections are indicated above each lane. The IP immunoblots shown.are representative of three independent infections and immunoprecipitations performed in each cell type. Note that cdk band intensities of gHV-cycV and gHV-cycK IPs are dramatically different from those of the mammalian cyclin expressing viruses, which, though consistent, are faint by comparison. (C) Viral cyclin localization is similar between gHV68 and KSHV viral cyclins during lytic infection. 3T12s were infected with WT, gHV-cycV and gHV-cycK viruses at an MOI of 5 PFU/cell and harvested at 12 and 24 hours post-infection. Cells were stained with anti-FLAG antibody and detected with Alexa Fluor 568 secondary antibody (red) and DAPI (blue). Original magnifications ×40. Representative images are shown from two independent experiments. (TIF) [file ppat.1002496.s002.tif]

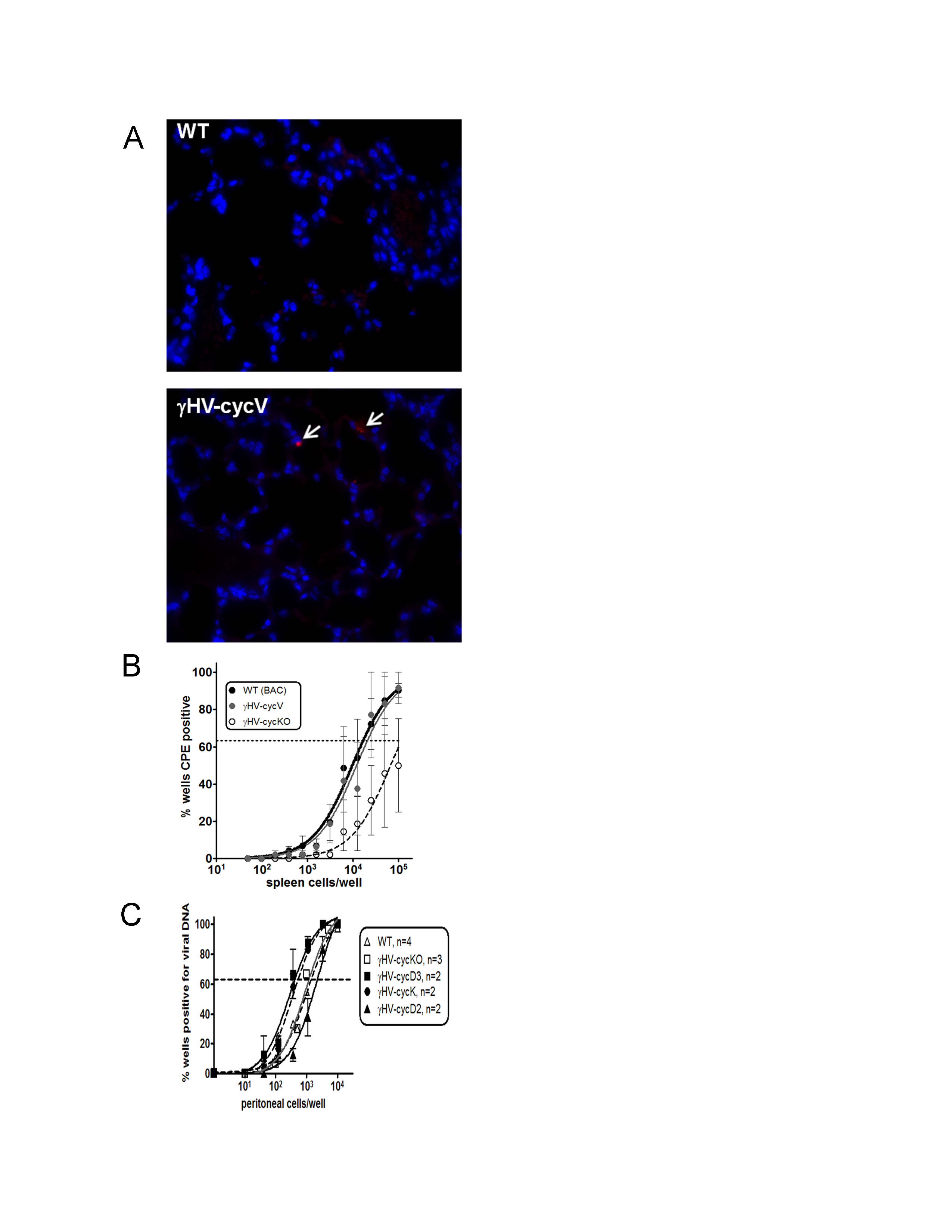

Supplement: Figure S3 — Cyclin recombinant viruses express 3x-FLAG cyclins and behave as expected during infection in vivo. (A) Viral cyclins are expressed during infection in vivo. Lung sections from C57BL/6 mice infected for 16 days with WT or gHV-cycV viruses were stained with anti-FLAG antibody and detected with Alexa Fluor 568 secondary antibody (red) and DAPI (blue). White arrows indicate positive FLAG staining, original magnifications ×40. Specific anti-Flag signal was detected in 3X-V-infected lungs at day 16 post-infection, with six Flag-positive cells counted in 12 fields of gHV-cycV infected lung examined, indicating approximately one cyclin-positive cell per 372 lung cells during establishment of latency. This is the first report of direct detection of the v-cyclin protein in infected tissue. (B,C) Limiting dilution analysis of latency (cyclin-independent) and reactivation (cyclin-dependent) of cyclin recombinant viruses following infection of C57BL/6 mice. Data represent the mean ± SEM of two-four independent experiments. (B) Frequency of spleen cells reactivating virus replication (wells resulting in cytopathic effect) from latency at 16 dpi. (C) Frequency of latently infected peritoneal cells (viral genome-positive cells) at 42 dpi. Frequency of latently infected cells was not statistically different among WT, gHV-cycKO, gHV-cycD3, gHV-cycK and gHV-cycD2. The dashed line at 63% indicates the value that was used to calculate frequency based on the Poisson distribution. (TIF) [file ppat.1002496.s003.tif]

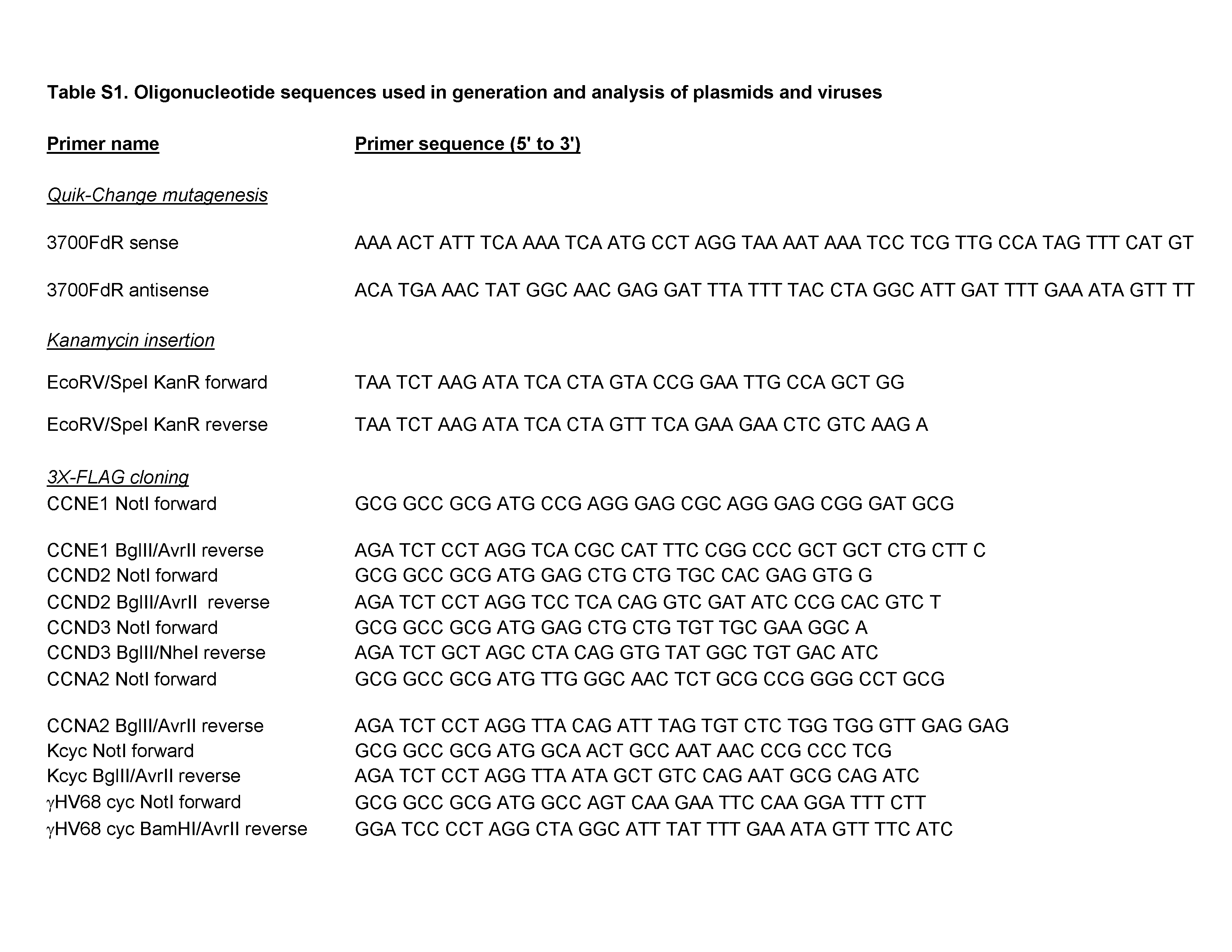

Supplement: Table S1 — Oligonucleotide sequences used in generation and analysis of plasmids and viruses. (TIF) [file ppat.1002496.s004.tif]
